# Silenced suppressor of cytokine signaling 1 (SOCS1) enhances the maturation and antifungal immunity of dendritic cells in response to *Candida albicans* in vitro

**DOI:** 10.1007/s12026-014-8562-8

**Published:** 2014-11-09

**Authors:** Dongmei Shi, Dongmei Li, Qingxin Yin, Ying Qiu, Hongxia Yan, Yongnian Shen, Guixia Lu, Weida Liu

**Affiliations:** 1 Department of Mycology, Institute of Dermatology, Chinese Academy of Medical Sciences and Peking Union Medical College, No. 12 Jiang Wangmiao Street, Nanjing, 210042 Jiangsu People’s Republic of China; 2 Department of Dermatology, Jining No. 1 People’s Hospital, Shandong, People’s Republic of China; 3 Jiangsu Key Laboratory of Molecular Biology for Skin Diseases and STIs, Nanjing, Jiangsu People’s Republic of China; 4 Georgetown University Medical Center, Washington, DC USA; 5 Shenzhen Second People’s Hospital, The First Affiliated Hospital of Shenzhen University, Shenzhen, Guangdong People’s Republic of China; 6 Anhui Medical University, Hefei, Anhui People’s Republic of China

**Keywords:** *Candida albicans*, Dendritic cells, Suppressor of cytokine signaling 1 (SOCS1), T helper 1 cells (Th1 cells), Interferon-γ

## Abstract

Dendritic cells (DCs) are known to play an important role in initiating and orchestrating antimicrobial immunity. Given the fact that candidiasis appears often in immunocompromised patients, it seems plausible that DCs hold the key to new antifungal strategies. One possibility to enhance the potency of DC-based immunotherapy is to silence the negative immunoregulatory pathways through the ablation suppressor of cytokine signaling suppressor 1 (SOCS1). Here, we deliver small interfering RNA (siRNA) against SOCS1 into murine bone marrow DCs, and as a consequence, we investigate the maturation/action of DCs and the subsequent T cell response after exposure to *C. albicans*. Our results show that the maturation of DCs (i.e., expressions of CD80, CD40, CD86, and MHC II) are significantly increased in the silenced SOCS1 treatment group after exposure to *C. albicans*. As a result, suppression of the SOCS1 promotes the greatest expression of IFN-γ and IL-12, and reduces IL-4 secretions, which induce CD4^+^ cell Th1 differentiation but inactivate Th2 cell development. The responses of IL-6 and TNF-β consist of up-regulation in the presence of *C. albicans*, but this is not specific to SOCS1 silencing, suggesting that these cytokines are not regulated by the SOCS1 gene in fungal infections. We find Th17 differentiation is unchanged regardless of SOCS1 inhibition. The increase in phagocytosis and killing of *C. albicans* in SOCS1 gene-treated DCs indicate a role for this cytokine suppressor in innate immunity as well. In conclusion, our findings support the view that SOCS1 protein is a critical inhibitory molecule for controlling cytokine response and antigen presentation by DCs, thereby regulating the magnitude of innate and adaptive immunities by generating IFN-γ-production T cells (Th1)—but not Th17—from naïve CD4^+^ T cells. Our study demonstrates that SOCS1 siRNA can serve as a useful vehicle to modulate the function of DCs against *C. albicans* infection.

**Electronic supplementary material:**

The online version of this article (doi:10.1007/s12026-014-8562-8) contains supplementary material, which is available to authorized users.

## Introduction

*Candida albicans* (*C. albicans*) is primarily commensal in healthy individuals, but under conditions of immune deficiency, this organism can cause a variety of infections with a high mortality rate that still presents challenges in clinical settings [1, 2]. Therapeutic strategies for candidiasis have gained several decades of new life with the development of azoles (a static antifungal agent) [3]. However, one of the consequences of relying heavily on azoles is the emergence of azole resistant strains that threaten to become nugatory for antifungal treatment. New therapeutic strategies have been promoted in a workshop sponsored by NIAID in 2006 [4]. One of the proposed new strategies involves modulating the host immune system to treat infectious disease. There are several potential advantages in this over the use of antifungals. First, it circumvents the problem of the rapid emergence of resistance. Second, this strategy offers the potential for a broad spectrum of activity against viral and fungal infections, bacterial infections and others. Third, immunomodulators of this type may benefit immunocompromised patients. Given the current high incidence rate of candidiasis in HIV/AIDS patients and the current propensity to treat those patients with fungistatic agents, this third benefit sounds particularly meaningful.

The host response for fungal infection includes innate and adaptive immunological mechanisms. The innate sensing mechanisms including dendritic cells (DCs) are usually hardwired to activate distinct CD4^+^ T cells to protect fungal infection. Over millions of years of evolution with commensal organisms like *C. albicans*, the typical host has developed an efficient network to fine-tune the balance between CD4^+^ effector T cells (Th1, Th2, or Th17) and T_reg_ cells, and between pro- and anti-inflammatory signals for either eradiation of fungi (limiting fungal burden) or tolerance of them (limiting damage to the host caused by the immune response) [5]. During this host response, DCs are adapted to govern the recognition of fungi and activate the different intracellular signaling pathways. The latter function is crucial for shaping T cell responses in adaptive immunity. For example, mature inflammatory DCs initiate Th1 and Th17 cell response in vivo following fungal stimulation via TLR adaptor MYD88, whereas immature tolerogenic DCs activate T_reg_ cell differentiation through signaling adaptor TRIF (TIR domain-containing adaptor protein) [6, 7].

Adaptive immunity has long been acknowledged as a major component of humans’ overall response to fungal infections. Distinct CD4^+^ T cell subsets coordinate protection against fungal disease, where Th1 and Th17 CD4^+^ T cell subsets promote fungal clearance and provide protective immunity against a variety of fungal pathogens [8, 9]. By contrast, Th2 cells have been found to play a detrimental role in most fungal disease. As we noted above, the differentiation of CD4^+^ T cells is largely dependent on their interaction with DCs and is modulated by cytokines produced by those DCs as well [10]. Therefore, the notion of being able to treat or prevent *C. albicans* infections through the manipulation of DCs maturation seems a practical approach.

It is well known that DCs are potent antigen-presenting cells and responsible for patrolling and securing the environment [11–13]. Upon detection and recognition of microbes or microbial components, DCs produce the cytokines and other molecules that can initiate the activation of proliferation and differentiation pathways of T cells [14]. A number of studies have shown that DCs are able to initiate and regulate the immune response to *C. albicans* and are crucial for defense against *C. albicans* infection in vivo [15]. Furthermore, a variety of DC-derived factors that induce T cell polarization have also been identified [5, 10]. DCs are usually divided into two subsets, tolerogenic immature and immunogenic mature cells according to their differentiation stages [16, 17]. The immature DCs have been recognized as able to produce small amounts of pro-inflammatory cytokines and large amounts of anti-inflammatory cytokines, which results in anergy, apoptosis of effector T cells, or induction and expansion of regulatory T cells [18, 19]. By contrast, mature DCs are able to secrete stimulatory cytokines and express high levels of co-stimulatory molecules that will stimulate the differentiation of CD4^+^ T helper cells and regulatory T cells in response to fungi cells and induce strong adaptive immunity via Th1, Th2, or Th17 effectors. Th1 CD4^+^ T cell differentiation is induced by IL-12 and IFN-γ, which leads in turn to the expression of the Th1 lineage-specific transcription factor T-bet to promote the fungal clearance process via IFN-γ [20]. Th17 cells are similarly induced by various cytokines such as IL-1β, TGF-β, and IL-6 [21, 22] and are responsible for recruitment of neutrophils upon the secretion of IL-17 and IL-22 [5]. Similar to T_reg_ cells, Th2 cells require IL-10 for their differentiation, but these T subset cells inhibit fungal clearance via IL-4 and IL-5 cytokines [5]. T_reg_ cells act mainly on anti-inflammatory activity in animal and human fungal infection via IL-10 and TGF-β that regulate or control the quality and magnitude of innate and adaptive effector response.

The suppressor of cytokine signaling 1 (SOCS1) has been discovered to be a critical inhibitory molecule for controlling the cytokine response and antigen presentation by DCs, thereby regulating the magnitude of both innate and adaptive immunity [23]. Previous studies suggested that lack of SOCS1 enhances the ability of DCs to activate naïve T cells [24] and induces stronger Th1-type responses both in vitro and in vivo in inflammatory disease and systemic autoimmunity [25]. SOCS1 is a key regulator of cytokine signaling that is important for maintaining the balance of immune responses. This signaling pathway also plays important roles in DC maturation through its negative cytokine signaling feedback loops.

Ablation of SOCS1 in antigen-presenting cells has been shown to enhance HIV-specific cellular immune response in mice [11]. Given that lack of SOCS1 leads to hyper-activation of DCs in HIV, we ask the question whether reducing expression of SOCS1 will facilitate an effective immune response against fungi such as *C. albicans.* To better understand the implication of SOCS1 on DCs and immune regulation in response to fungal infection, we utilize murine bone marrow DCs to characterize the immune response by means of siRNA-mediated reduction in SOCS1 mRNAs. As we expected, the abolition of SOCS1 in murine bone marrow DC results in enhanced DC maturation and phagocytosis capability. The cytokine profile in the presence of fungal cells reveals that the immune response favors the differentiation of Th1 T cells, but not the differentiation of Th17, which should better enable the subsequent clearance of *C. albicans*.

## Materials and methods

### Mice

Female C57 mice were purchased from the Experimental Center of Yangzhou University (Jiangsu, China) for all animal experiments. The mice were bred in the animal experimental center of Institute of Dermatology, Chinese Academy of Medical Sciences (CAMS) in Nanjing, Jiangsu. All the mice are at age of 8 weeks at the start of investigation, and the body weight was in the ranges of 16–18 g. The experiments were performed in a pathogen-free environment. The protocols of all animal experiments were approved by the Animal Study Committee of the Institute of Dermatology, CAMS, according to the government guidelines for animal care.

### DCs preparation

Bone marrow-derived DCs were isolated and maintained as described earlier with minor modifications [26]. Briefly, bone marrow cells were obtained from femurs and tibias of C57 mice, filtered through nylon mesh. RBC were depleted with lysis buffer and washed with PBS twice. Then, the cells were seeded at 2 × 10^6^ cells per 100-mm dish in RPMI 1640 medium supplemented with 10 % fetal bovine serum (FBS) (Gibco, USA) and 200 U/ml rmGM-CSF (Propetech, USA). At day 2, 5 ml of RPMI 1640 medium containing 200 U/ml rmGM-CSF was added to the plates. At days 4, 6, and 8, half of the culture supernatants were collected and centrifuged, respectively. Each cell pellet was re-suspended in a 5 ml of a fresh RPMI 1640 medium containing 200 U/ml rmGM-CSF and returned to the original plate. At day 8, the DCs were harvested for subsequent experiments. Flow cytometric analyses confirmed that more than 95 % cells in the culture plate were CD11c^+^ cells.

### Synthesis of siSOCS1 gene and gene transfection

The siRNAs targeting the murine SOCS1 gene were designed using online siRNA programs: siRNA Selection Program (http://sirna.wi.mit.edu/home.php), siDirect version 2.0 (http://sidirect2.rnai.jp) and Deqor v3 (http://deqor.mpi-cbg.de/deqor_new/input.html). The siRNAs molecules used for suppression of murine SOCS1 gene and the negative control siRNA (which does not target any sequence present in the murine genome) were all obtained from Hangzhou Biosci Co., Ltd. The sense strand sequences of siRNA target sites designed to silence the murine SOCS1 gene were as follows: siSOCS1 #1 (5′-TCCGCACCTTCCGCTCCCA-3′), siSOCS1 #2 (5′-ACACTCACTTCCGCACCTT-3′), siSOCS1 #3 (5′-ACTTCCGCACCTTCCGCTC-3′), and control siRNA (5′-CAGCCTTCCTTCTTGGGTAT-3′). Recombinant lentivirus expression vector GV248 was used as the vector for SOCS1 siRNA. Transient transfection of siRNA was carried out with the lipofectamine 2,000 regent (Invitrogen) according to the manufacturer’s instructions.

### Quantitative RT-PCR

For gene silencing experiments, lipofectamine with 400 pmol of siSOCS1 (molar ratio 10:1) was applied to 4 × 10^5^ DCs with each siRNA molecule. The relative expression of SOCS1 mRNA in trans-infected DCs was evaluated by quantitative real-time PCR (qRT-PCR). Total RNA was prepared using TRIzol reagent (Invitrogen) according to the manufacturer’s instructions. cDNA was synthesized with 1 μg of total RNA by reverse transcriptase (Takara). For quantitative determination of SOCS1 expression, qRT-PCR analysis was performed with LightCycler (Roche Diagnostics). The following primers were used in qRT-PCR: SOCS1 (forward, 5′-GCCATCTTCACGCTGAGC-3′; reverse, 5′-GCCATCTTCACGCTAAGGGC-3′); and β-actin was used as an internal control with primers as follows (forward, 5′-CAGCCTTCCTTCTTGGGTAT-3′; reverse, 5′-CTGTGTTGGCATAGAGGTCTT-3′).

### Western blot analysis

In western blot analysis, cell lysates were separated by SDS-polyacrylamide gel electrophoresis, transferred onto polyvinylidene fluoride (PVDF) membranes, and probed with rabbit monoclonal anti-SOC1 antibody (1:1,000 dilutions; A156; #3950, Cell Signaling Technology, Boston, USA). Bound antibodies were detected using a horseradish peroxidase (HRP)-labeled goat anti-rabbit IgG and then were developed using 3,3′,5,5′-tetramethylbenzidine (TMB).

### Cytotoxicity assay

To observe whether SOSC1 silencing induces apoptosis or necrosis in DCs, we measured the staining capacity of an FITC-conjugated monoclonal antibody (mAb) against activated caspase-3 (BD Bioscience, USA). Briefly, the DCs with siSOCS1 silencing were incubated in Cytofix/Cytoperm solution at a concentration of 2 × 10^6^ ml for 20 min on ice. Following re-suspension in 100 μl of Perm/Wash buffer (BD Bioscience, USA), the cells were incubated with 20 μl of FITC-conjugated caspase-3 mAb for 30 min in the dark at room temperature and then analyzed by a flow cytometry. DCs from the same experiments were also stained with trypan blue (Sigma, USA) to determine the cell viability.

### DCs phagocytosis

DCs with SOCS1 silencing or not were collected, respectively, washed, and seeded in 96-well plates at 5 × 10^5^ in 100 μl per well for 2 h. Then, cells collected from an overnight culture of *C. albicans* (1 × 10^8^) were labeled with fluorescein isothiocyanate (FITC, Sigma, 1.25 mM in 0.1 mM sodium bicarbonate buffer with 0.5 % DMSO, pH 9.0) and maintained at 4 °C for the entire overnight period. After 3 times of PBS washes to remove unbound dye, labeled yeast cells suspended in 100 μl of DMEM + 10 % FBS and mixed with DCs at a ratio of DC:yeast = 1:1. The plates were incubated for 1 h at 37 °C in a 5 % CO_2_ atmosphere. At each time point, 100 μl of trypan blue (250 mg/ml in PBS) was added to quench the fluorescence of yeasts that were bound but not internalized at room temperature for 1 min. The cells were collected and analyzed by flow cytometry. Meanwhile, image of internalized and fluorescent yeasts was visualized by fluorescence microscopy.

### DCs killing assay

DCs were prepared and seeded in a 96-well plate at 5 × 10^4^ in 150 μl of RPMI 1640 for 12 h at 37 °C as described above. The overnight cultures of fungal strains were diluted into DMEM at 2 × 10^6^ ml/l. A 50 μl-aliquot was added into the first column of the plate, to mixed with DCs, and then serially diluted at 1:4 for 6 times. Strains incubated without DCs were diluted in parallel manner. All the plates were incubated for 24 h at 37 °C in a 5 % CO_2_ atmosphere. Colonies were counted by microscopy in each well. The survival rate of each strain was calculated as the numbers of colonies with DCs divided by the number of colonies in the absence of DCs × 100 % [27]. Each experiment was duplicated and repeated three times.

### Flow cytometric analysis of surface antigen expression of DCs infected with *C. albicans*

DCs were co-cultured with *C. albicans* as described above. Then, DCs were collected, and expression of surface molecules on DCs was quantified by flow cytometry using FITC- or PE-conjugated Ab (anti-CD11c, anti-I-A/anti-I-A/I-E, anti-CD40, anti-CD80, and anti-CD86) (eBioscience Inc., San Diego, CA, USA) and carboxyfluorescein-conjugated rat anti-mouse CCR7 (4B12) mAb (R&D, USA). The samples were analyzed using a FACSCalibur flow cytometer and CellQuest software (FACSCalibur, Becton–Dickinson, USA) was used.

### Measurement of cytokine secretion by DCs infected with *C. albicans*

The expression of cytokines was measured by ELISA. DCs were prepared as described above. Briefly, DCs (5 × 10^4^ in 100 μl) were infected with 100 μl of yeast cells (1 × 10^4^) in RPMI 1640 supplement with 10 % FBS and co-cultured at 37 °C in a 5 % CO_2_ atmosphere for 24 h. As a negative control, DCs were cultivated without yeast. After centrifugation of the plates at 4 °C for 15 min at 2,250*g*, the supernatants were collected and stored at −80 °C until assays were performed. The expression of IFN-γ, IL-12, IL-4, TGF-β, IL-10, and IL-6 secretion was measured by ELISA kit according to the manufacturer’s protocol. Background levels for non-infected DCs were measured to obtain final determinations of cytokines levels of co-cultivated cultures.

### T cell proliferation assays

CD4^+^ T lymphocytes were isolated from spleen of C57 mice by negative immunomagnetic using a CD4^+^ T cell isolation kit II (Miltenyi Biotec, Bergisch Gladbach, Germany). Cell purity was assessed by flow cytometry using PE-conjugated anti-CD4 mAbs (eBioscience Inc., San Diego, CA, USA). The purity reached to >95 % in the cell population.

To trace proliferation, T cells were labeled with 10 μm CFSE (Invitrogen, Grand Island, NY, USA) according to the manufacturer’s instructions [28]. For these assays, spleen CD4^+^ T cells isolated from 6 of C57 mice were first incubated with 10 μM CFSE (Invitrogen, Carlsbad, CA) at 37 °C for 8 min. Meanwhile, DCs were incubated for 24 h at 37 °C with *C. albicans* and then washed, co-cultured with the CFSE-labeled allogeneic T cells at different DCs/T cell ratios (0, 1:10, 1:20, 1:40, and 1:80) in RPMI containing 5 % FCS for 2 days at 37 °C in triplicate. After 2 days of incubation, T cell proliferation status was examined by formation of T cell clusters under a microscope and CD4^+^ T cells were verified by flow cytometric measurement with anti-CD4 mAb (BD Biosciences).

### Determination of cytokine production by CD4^+^ T cells

Highly purified CD4^+^ T cells were isolated as described above. Purified CD4^+^ T cells (1 × 10^5^) were co-cultured with 1 × 10^4^ irradiated DCs (30 Gy). Recombinant murine anti-IL-12p70 or/and anti-INF-γ (R&D Systems, USA) at a concentration of 10 ng/ml were added to T cell cultures that have been stimulated with DCs following exposure to SOCS1 siRNA. After 48 h, cells were activated with phorbol myristate acetate (50 ng/ml) and ionomycin (50 ng/ml) in the presence of brefeldin A (10 µg/ml) for 6 h. For intracellular cytokine production analysis, cells were harvested, washed, fixed, and permeabilized using the CytoStain kit (BD Pharmingen, USA) according to the manufacturer’s instructions. Cells were then stained with 0.5 µg/test of anti-murine IL-4-PE, anti-murine-IFN-γ FITC, and anti-murine IL-17 (BD Pharmingen, USA) and were analyzed by flow cytometry. IFN-γ, IL-4, and IL-17 production by CD4^+^ T cells was also assessed using the Cytometric Bead Array kit from BD Pharmingen.

### Statistical analysis

Statistical analyses were performed using SPSS 16.0. Based on these software determinations, the quantitative differences between sample groups were determined by either one-way ANOVA followed by a Tukey test or independent samples *t* test. Data were presented with the mean ± standard deviation (SD). Statistical significance was defined as *p* value <0.05.

## Results

### Experimental validation of miRNA targets

The viability of DC cells following siRNA treatment was tested first. The DCs with SOCS1 siRNA or control vector GV248 (recombinant lentivirus expression vector) treatments were stained with activated caspase-3, a marker of early apoptosis. We found that <2 % of DC population was stained positively with FITC-conjugated caspase-3 mAb after SOCS1 siRNA treatment or exposure to GV248. The results indicated that SOCS1 siRNA and GV248 were not toxic to DCs (Supplemental Fig. 1a–c) Trypan blue exclusion also confirmed that DCs were viable after infection by SOCS1 siRNA or GV248 (Supplemental Fig. 1d). In order to determine whether inhibited effects of SOCS1 gene are in fact operating in DC cells, the SOCS1 protein and gene expression levels were measured by both western blot analysis and qRT-PCR. For three target transfections (SOCS1 siRNA #1, #2, and #3), SOCS1 protein levels reduced to 25–75 % of their original level in DCs, and SOCS1 siRNA #2 infection in particular was most effective, exhibiting a 75 % reduction. This result was consistent with SOCS1 mRNAs levels in treated DC cells. In supplemental Fig. 2, the gene expression in SOCS1 siRNA #2 transfected DCs was down-regulated by 75 % after normalization with reference gene β-actin. Thereafter, SOCS1 siRNA #2 infected DCs were used in all subsequent experiments in this study.

### Ablation of SOCS1 siRNA increased phagocytosis and killing capacity of DCs

**Fig. 1:**
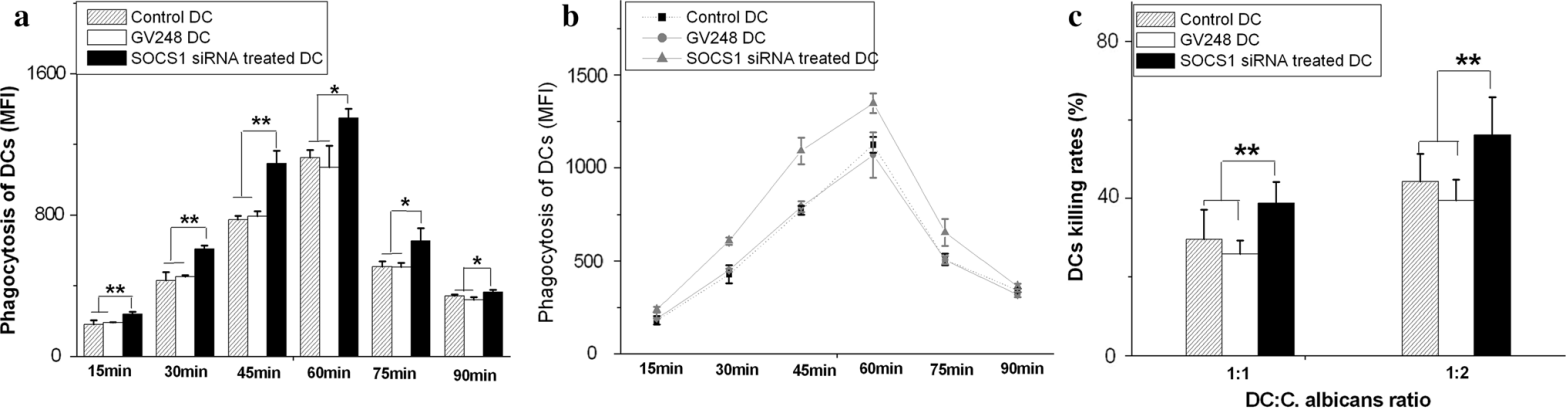
SOCS1 enhanced the phagocytosis and killing of *C. albicans* by DCs in vitro. Untreated DCs served as the control. **a**, **b** DC was co-cultured with *C. albicans* labeled with FITC at a ratio of DC:yeast = 1:1. The portion of DC ingesting FITC staining *C. albicans* was observed under a fluorescence microscope (×200). A significant enhancement of phagocytosis in SOCS1 siRNA-treated DCs was determined (*p < 0.05). **c** Viability of *C. albicans* is shown after an overnight incubation with DCs. Viable colonies of each strain were counted. Data are averages of seven separate experiments. *p < 0.05; **p < 0.01

Phagocytosis of DCs plays a critical role in defending the host against the pathogen [29]. To determine the role(s) of SOCS1 in innate immunity in the presence of fungi, we proceeded to test whether the capacity of DC phagocytosis of *C. albicans* was altered by SOCS1 silencing. Compared with control DCs, DCs infected with SOCS1 siRNA had a significantly higher phagocytosis rate (Fig. 1a). The phagocytosis capacity reached its peak when DCs co-cultured with *C. albicans* at 60 min (Fig. 1b). As the co-cultured time increased, however, DCs phagocytosis declined, which may be due to maturation of DCs after stimulated by pathogens. We also compared the killing capacities of control DCs to that of SOCS1 siRNA-treated DCs after 24 h of incubation with *C. albicans*. SOCS1 interfered DCs had a higher killing rate than that of control DCs (Fig. 1c). Our data suggested that SOCS1 silencing in DCs promoted their phagocytosis and killing abilities.

### SOCS1 siRNA effectively enhances the maturation of DCs in response to *C. albicans*

**Fig. 2:**
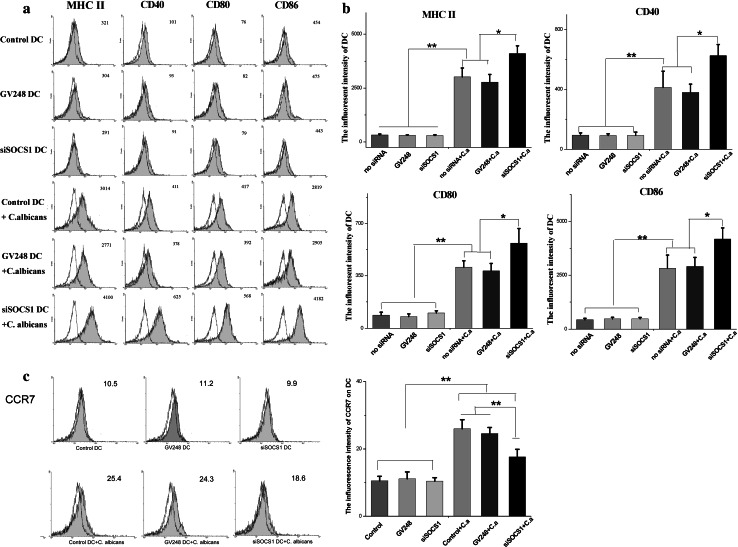
DCs treated with SOCS1 siRNA have a higher maturation phenotype. Immature DCs were exposed to medium alone, or treated with SOCS1 siRNA, and then, *C. albicans* was added for 24 h to induce maturation. Surface phenotype on DCs was analyzed by flow cytometry. **a**, **c** Expression of maturation and co-stimulatory molecules. Representative data from one of the seven experiments are shown. The mean fluorescent intensity (MFI) corresponding to the expression of surface antigen is indicated in each histogram. **b** Mean ± SD of data from seven independent experiments is shown. *X-axis* represents MFI. DCs were exposed to medium alone or SOCS1 siRNA for 2 h. Some DCs were treated with *C. albicans* for at least 24 h. *p < 0.05; **p < 0.01

To determine if SOCS1 siRNA plays a role in modulating the maturation of DCs in response to *C*. *albicans*, the activation of a number of cell surface markers was analyzed by flow cytometry. We found that cultured DCs exhibited typical immature phenotype at first (such as MHC II low, CD40 low, CD80 low, and CD86 low) but underwent maturation after treatment with *C*. *albicans*, as evidenced by high expressions of MHC II, CD40, and co-stimulatory molecules such as CD80 and CD86. In the absence of *C. albicans,* low levels of these cell surface molecules were detected in SOCS1 siRNA-treated DCs that were similar to those of control DCs and vector treated DCs in Fig. 2a. However, after each group of DCs was co-cultured with *C. albicans* cells, the expression of these four cell molecules on DCs surfaces showed a significant increase when compared to DCs without fungal cell stimulation (Fig. 2b). The activation rate of SOCS1 siRNA-treated DCs was even higher (by an additional 25 %) when compared with untreated DCs and vector treated DCs. These results indicate that suppression of SOCS1 promotes DC maturation when accompanied by *C. albicans*.

DC maturation leads to profound changes in antigen uptake, processing, and presentation capabilities. Apart from those molecules mentioned above, chemokine receptor 7 (CCR7) is also up-regulated upon DC maturation. CCR7 acts together with its chemokine ligands (CCL19 and CCL21) to mediate the migration of immune cells to secondary lymphoid organs including DCs [30]. The physical contacts between subpopulations of T cells and DCs in these secondary lymphoid organs are then necessary for the optimal initiation of protective immunity. To characterize the CCR7 profile in SOCS1 silenced DCs, we also examined the expressions of CCR7 on different DC groups. We observed that CCR7 was barely evident in both infected and untreated DC cells in the absence of *C. albicans*. But the up-regulation of CCR7 occurred in both control DCs and SOCS1-slienced DCs after co-culture with *C. albicans*, although the expression level of CCR7 on SOCS1-silenced DCs was not as high as that of control DCs. Unlike MHC II, CD40, CD80, and CD86, the increases in CCR7 levels were triggered by *C. albicans*, but not by SOCS1 inhibition, as we show in Fig. 2c.

### Cytokine production in SOCS1 siRNA-treated DCs in response to *C. albicans* stimulation

**Fig. 3:**
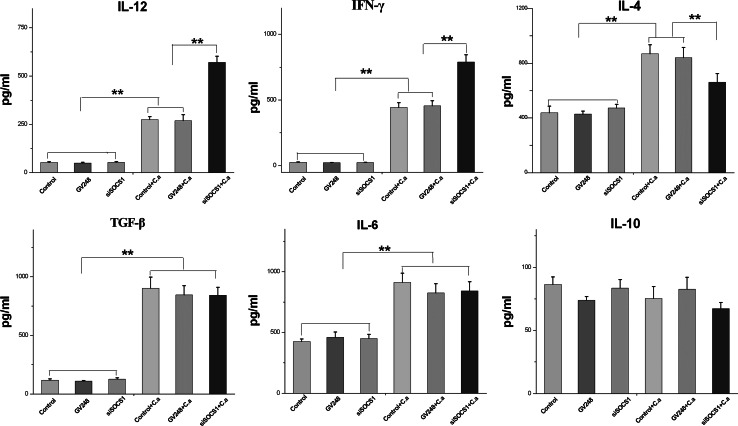
DCs treated with SOCS1 siRNA produce high levels of IL-12 and IFN-γ and low level of IL-4, but have no effect on the production of IL-10, IL-6, and TGF-β. At day 8, iDCs were exposed to SOCS1 siRNA and treated with or without *C. albicans* for 24 h, and then, culture supernatants were collected. The concentration of cytokines was measured using ELISA kits. Mean ± SD of seven experiments is shown. *p < 0.05; **p < 0.01

To fully understand how cytokines respond with DC SOCS 1 gene inhibition in the presence of *C. albicans*, we next examine the effect of SOCS1 siRNA on cytokine production by measuring pro-inflammatory cytokines such as IFN-γ, IL-12, IL-4, IL-10, IL-6, and TNF-β. These cytokines induced or produced by DCs in response to *C. albicans* have been demonstrated to be important mediators that control later T cell response. For example, IL-12 is the major inducer of Th1 cells producing IFN-γ on undifferentiated T cells, and IL-10 is more favored in Th2 or T_reg_ differentiation. As the results show in Fig. 3, the baselines levels of IL-12, IFN-γ, and TNF-β were no different and maintained low concentrations in each group of DCs in the absence of *C. albicans* stimulation. However, expression of these three cytokines in siRNA-treated and untreated DCs increased significantly after 24 h of incubation with *C. albicans* cells. The quantity of each cytokine was 4–5 times higher in each exposed group of DCs than in the respective group of unexposed DCs, with the highest levels of IL-12 and IFN-γ being produced by SOCS1 siRNA-infected DCs. On the other hand, the overall levels of IL-4 and IL-6 increased only twofold in each DC group after *C. albicans* stimulation. Although no difference has been noted among three groups of DCs for IL-4 and IL-6 without *C. albicans*, we found that SOCS1 siRNA-infected DCs produced less IL-4 when compared with control DCs in the presence of *C. albicans* cells, implying a negative regulation of this cytokine during *C. albicans* infection. Intriguingly, the cytokine IL-10 profiles were similar to each other regardless of SOCS1 siRNA treatment or *C. albicans* stimulation in this study.

In summary, SOCS1 siRNA-treated DCs incubated with *C. albicans* produced higher levels of IL-12 and IFN-γ but lower levels of IL-4 than the control DCs. Although TGF-β and IL-6 increased after fungal cells stimulation, no significant differences were observed between SOCS1 siRNA-treated DCs and control DCs in regard to TGF-β, and IL-6 production and IL-10, indicating that these three cytokines are specific to *C. albicans* infection and are not regulated by SOCS1 gene. The results obtained through immunostaining of the intracellular cytokine are consistent with these observations (data not shown).

### SOCS1 siRNA-treated DCs had enhanced ability to stimulate CD4^+^ T lymphocyte proliferation

**Fig. 4:**
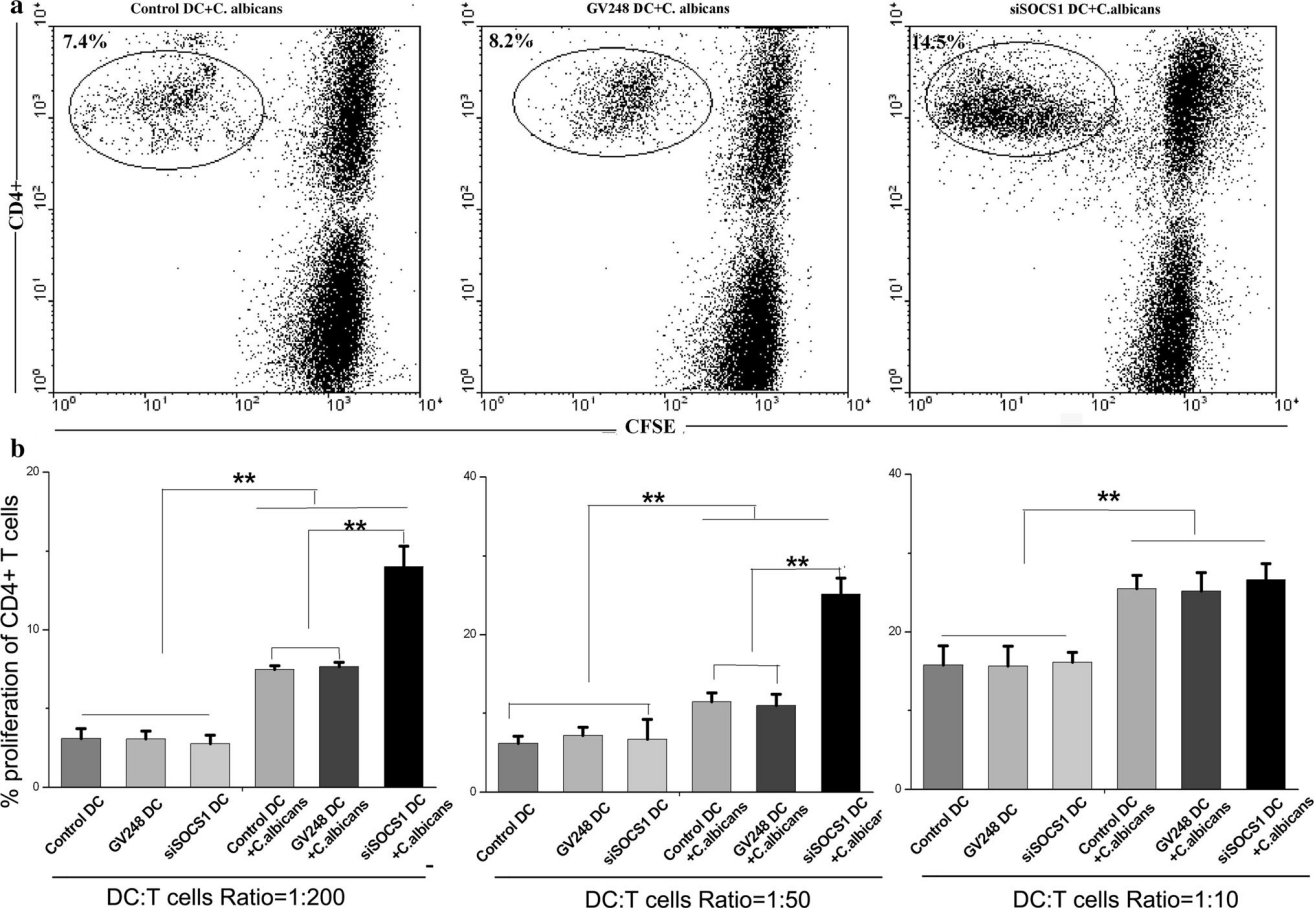
DCs treated with SOCS1 siRNA have enhanced capacity to activate T cells. Purified T cells were counted and seeded into 96-well plates at 10^5^ cells/well at room temperature with an equal volume of 2.5 μm CFSE. DCs were exposed to medium alone or SOCS1 siRNA, treated with *C. albicans* for 24 h, to stimulate T cells. Five hundred, 2,000, or 10,000 irradiated DCs were added to triplicate wells. After 5 days, the cells were collected and stained with PE-conjugated anti-CD4 mAbs for analysis. **a** The numbers in the *upper left of each graph* represent the percent of CD4^+^ T cells that was proliferating. **b** The results are expressed as the percent of CD4^+^ T cells at different ratios of DC-to-T cells. Mean ± SD of data from seven experiments is shown. **p < 0.01

The bipolar nature of the immune-related inflammatory process in fungal infection has been demonstrated by clinical manifestations of chronic mucocutaneous and disseminated candidiasis. The inflammation that is driven by cytokines and T effectors is beneficial to the infection in most circumstances, but an uncontrolled inflammatory response is detrimental and may eventually oppose disease outcome. As we mentioned above, SOCS1 gene is an important regulator that suppresses DC maturation and cytokine production during inflammatory response. To clarify the ability of T cell differentiation under SOCS1 gene silencing, we compared the proliferation of allogeneic CD4^+^ T cells after incubation with SOSC1 siRNA DCs or control DCs. Immature DCs were co-cultured with *C. albicans* for 24 h prior to stimulation of allogeneic T cells. As shown in Fig. 4, SOCS1 siRNA-silenced DCs had an enhanced capacity to stimulate the proliferation of allogeneic T lymphocytes at DC-to-T-cell ratios of both 1:50 and 1:200. By contrast, no activation of T lymphocytes was seen in untreated DCs. This implicates that SOCS1 siRNA-treated DCs are implicated in T cell differentiation. However, when the DC-to-T-cell ratio was increased to 1:10 or greater, allogeneic T cell proliferation was similar to the level that was achieved by control DCs. Our explanation for this nonlinear dose response is that it is most likely due to high levels of surface molecules such as MHC II and other co-stimulatory elements in SOCS1 siRNA-treated DCs as we described previously. Perhaps, once the level of the surface molecules and the concentration reaches saturation in DCs, the proliferation of T cell is no longer affected by the number of DCs.

### SOCS1 siRNA-treated DCs showed enhanced ability to induce IFN-γ producing CD4^+^ T cells

In conjunction with the significant increase of IL-12 and IFN-γ in cytokines analysis described above, T cell polarization was also assessed in the SOCS1 siRNA DCs to determine whether Th1 polarization was affected in SOSC1 siRNA-treated DCs after co-culture with *C. albicans*. In this experiment, SOCS1 siRNA-treated DCs were exposed to *C. albicans* and then were used to stimulate purified CD4^+^ T cells. As noted above, allogeneic CD4^+^ T cell proliferation was increased when stimulated at a high ratio of SOCS1 siRNA-treated DCs to T cells (1:50) when compared with control DCs.

**Fig. 5:**
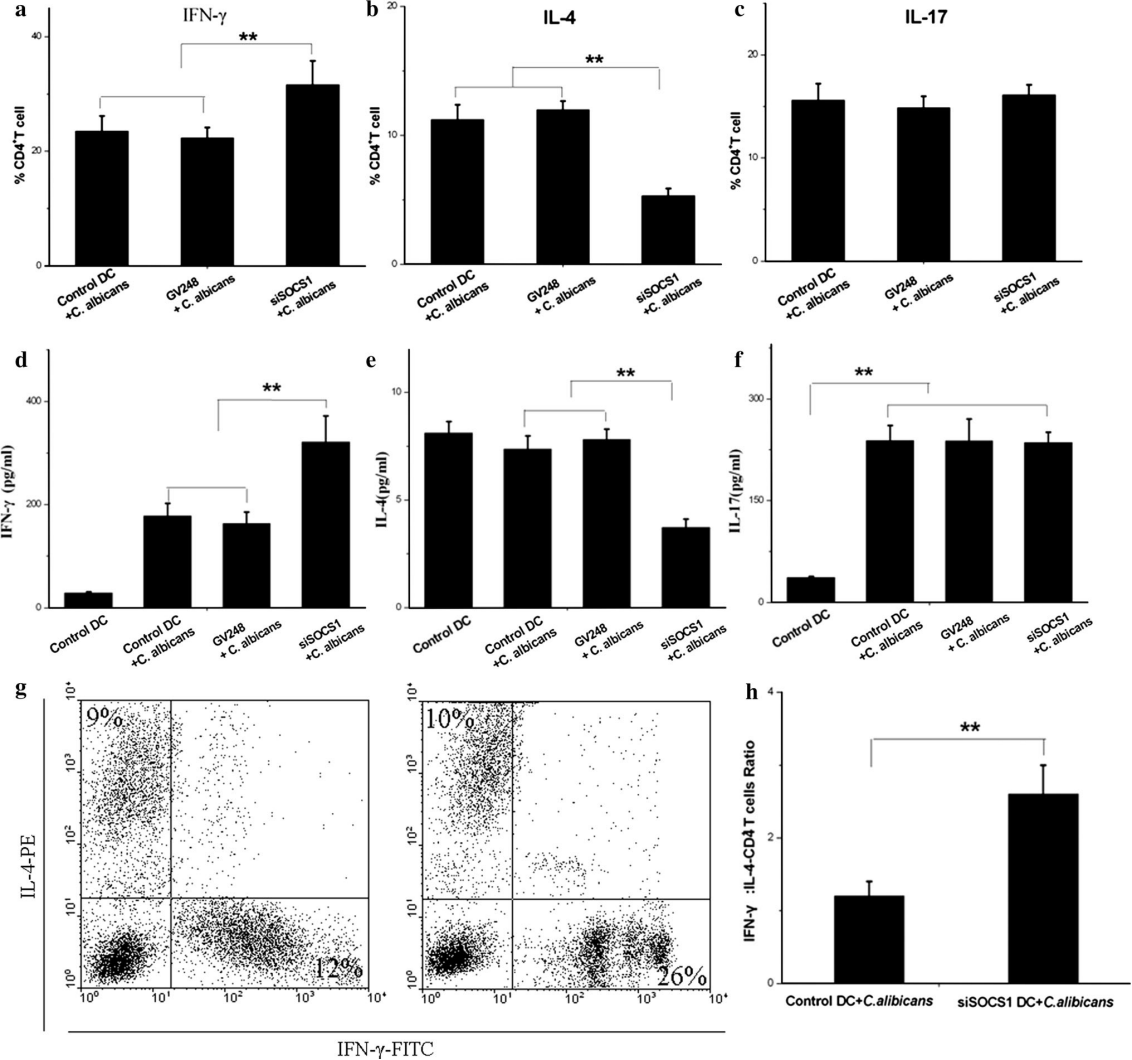
SOCS1 siRNA treatment DCs co-cultured with *C. albicans* induced strong Th1 responses in MLR. Proliferative capacity of allogeneic T cells from C57 mice co-cultured with DCs in MLR. **a**–**c** IFN-γ, IL-4, and IL-17 concentrations in the culture medium were measured by flow cytometry. Cells were stained with anti-IL-4-PE, anti-IFN-γ-FITC, and IL-17-FITC to detect intracellular cytokine production by CD4^+^ T cells. Mean ± SD of data from seven independent experiments is shown. **d**–**f** Supernatants were collected on day 5 of the culture, and IFN-γ, IL-4, and IL-17 concentrations analyzed using cytometric bead arrays. Mean ± SD of data from seven independent experiments is shown. **p < 0.01

IFN-γ, IL-4, and IL-17 productions by T cells are hallmarks of Th1, Th2, and IL-17 responses, respectively [31]. The proportion of CD4^+^ T cells producing IFN-γ, IL-4, or IL-17 was determined by intracellular cytokine staining in Fig. 5a–c. The products of these three cytokines were measured by ELISA as shown in Fig. 5d–f. Compared with control DCs, DCs treated by SOCS1 siRNA generated a higher population of IFN-γ-producing CD4^+^ T cells but lower populations of IL-4-producing CD4^+^ T cells (*p* < 0.05). Accordingly, secretion of IFN-γ and IL-4 in CD4^+^ T cells were notably enhanced and reduced, respectively. On the other hand, the increase of IL-17 level in the presence of *C. albicans* did not correlate with Th17 population since the number of IL-17-producing CD4^+^ T cells remained the same when treated with SOCS1 siRNA DCs.

The ratios of IFN-γ producing CD4^+^ T to IL-4-CD4^+^ T cells induced by SOCS1 siRNA DCs and control DCs after exposure to *C. albicans* were 2.6 ± 0.4 and 1.2 ± 0.2, respectively (*p* < 0.002) in Fig. 5h, further highlighting the effect of SOCS1 siRNA DCs on T cell polarization. With its modest effect on IL-4 production (Fig. 5), SOCS1 siRNA treatment enhanced capacity of DCs to induce IFN-γ-producing CD4^+^ T cells differentiation significantly, even at a high DC-to-T cell ratio, strengthening our hypothesis that SOCS1 siRNA modulates the immune response and CD4^+^ T cells polarization by modifying the function of DCs.

### Exogenous anti-IL-12 or anti-IFN-γ mAbs reversed the enhancement of INF-γ production by CD4^+^ T cells

**Fig. 6:**
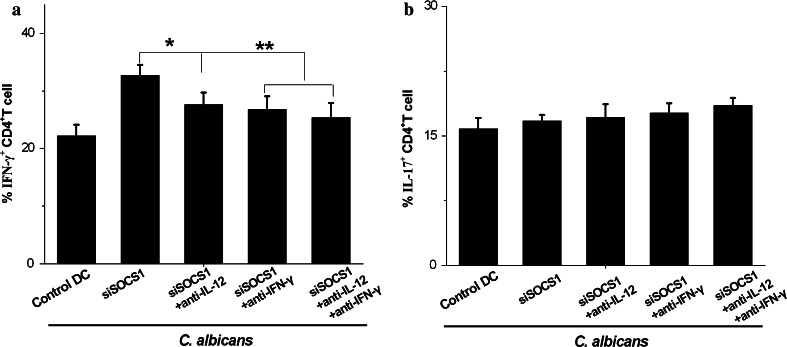
Exogenous anti-IL-12 or anti-IFN-γ mAbs reversed the enhancement of IFN-γ production by CD4^+^ T cells. **a**, **b** DCs were exposed to SOCS1 siRNA and were treated with *C. albicans* for 24 h. At day 10, T cells were stimulated with PMA and ionomycin for 5 h in the presence of brefeldin A. Cells were stained with anti-IFN-γ-FITC and IL-17-FITC to detect intracellular cytokine production by flow cytometry. Data were Mean ± SD of seven independent experiments. *p < 0.05; **p < 0.01

Th1 cell development is dependent on the presence of IL-12 and IFN-γ, and on the ability of T cells to respond to IL-12 and IFN-γ. Here, we propose to validate the hypothesis that SOCS1 siRNA treatment promotes Th1 polarization in the presence of *C. albicans* through these two cytokines, and to answer whether this up-differentiation arises from direct cellular contact (DC-T cells) or soluble factors (IL-12 or IFN-γ) or both. In the co-cultured experiments described above, we attempted to reverse this T cell differentiation by the addition of saturating amounts of anti-IL-12 or anti-IFN-γ mAbs. The results showed that each antibody alone had only moderate effects on CD4^+^ Th1, but addition of both anti-IL-12 and anti-IFN-γ mAbs to the co-cultures reversed the enhancement far more completely, and also persisted longer, as shown in Fig. 6a. Furthermore, these two antibodies had no effect on the population of Th17 cells in Fig. 6b. These results suggest that pro-inflammatory cytokines (IL-12 and IFN-γ) are significant contributors to CD4^+^ T cell differentiation when co-cultured with SOCS1-silenced DCs. However, such inhibition effects were not total, even after treatment with both anti-IL-12 and anti-IFN-γ mAbs when compared with baseline levels (see Fig. 6a).

## Discussion

Immune therapies for fungal infection still present significant challenges today. The several breakthroughs in our understanding of how immune mechanisms perform in *C. albicans* mucosal colonization and bloodstream invasion should guide us in the invention of new therapies beyond simple antifungal. DCs are the most potent antigen-presenting cells in the immune system and have the unique ability to take up and efficiently present antigens to naive T lymphocytes. DCs can also interact with B cells and natural killer (NK) cells, thus bridging the gap between innate and adaptive immunities. In this study, we demonstrate that SOCS1 siRNA approach can be used to silence negative regulatory molecules in DCs and thereafter to modulate immune response. Our data show that reversing the immunity-attenuating mechanism of SOCS1 activates the function of DCs, including the promotion of DC maturation and the regulation of pro-inflammatory cytokines such as IL-12, IFN-γ, and IL-4 in presence of *C. albicans*, which in turn activate Th1 T cell differentiation. At the same time, SOCS1-ablated DCs also mediate innate immunity by having a stronger phagocytosis and killing capacity. These results suggest that the targeted modulation of SOCS1 expression in DCs could be exploited as a novel molecular adjunct to improve the potency of vaccine-induced Th1 cell responses to *C. albicans* infection.

*C. albicans* is an opportunistic fungal pathogen that normally exists as a harmless commensal in humans, but in immunocompromised individuals, *Candida spp.* can also cause disseminated infections that have a high mortality rate and represent a major problem in clinical settings [32, 33]. Enhancement of immune system efficacy can be an innovative strategy to prevent or treat *C. albicans* infection. DCs play an important role in initiating and orchestrating antifungal immunity and also participate in recognizing and eliminating fungal pathogens [34, 35]. DC-based approaches are being intensively investigated as potential preventive and therapeutic strategies for several diseases including pathogenic infection disease [13, 36]. In addition to the expression of co-stimulatory molecules that facilitate an immune response, DCs are also equipped with negative feedback mechanisms that control their cytokine function.

In this study, we compare SOCS1 siRNA-treated DCs with control DCs, and we find that the former display even higher expression of MHCII and cell surface molecules such as CD40, CD80, and CD86 (which are common indicators of the maturation of DCs) following stimulation by *C. albicans.* Although the ablation of SOCS1 in DCs could by itself induce the expression of co-stimulatory molecules as suggested by others, we find that there was no significant difference in surface molecule expression between SOCS1-treated DCs and control DCs that did not undergo pathogen stimulation. These results suggest that the DC maturation process is activated by SOCS1 inhibition.

Phagocytes such as DCs and macrophages employ phagocytosis by taking up pathogens (such as bacteria or fungi) into phagosomes, which digest the pathogens and then present the bacteria- or fungi-derived peptide antigens to the host’s adaptive immunity [37]. Therefore, efficient antigen presentation depends greatly on a well-regulated phagocytosis process. Our results show that SOCS1 silencing enhances phagocytic uptake and the killing capacity of *C. albicans* by DCs. Our findings also are consistent with the prevention and treatment of pathogenic infections such as those from fungi.

Ablation of SOCS1 in antigen-presenting cells has been reported to enhance cellular immune response and subsequent cytokine responses in mice when they are challenged with a pathogen [38]. The SOCS1-silenced DCs stimulated by *C. albicans* in this study also show high levels of cytokine expression, including IL-12 and IFN-γ, which orchestrate the induction of the strong CD4^+^ Th1 responses necessary for effective cell-mediated adaptive immunity to intracellular pathogens. However, the cytokines such as TGF-β and IL-6 produced by SOCS1-silenced DCs stimulated by *C. albicans* (which presumably sustain T_reg_ and Th17 differentiation and maintenance [15, 22, 39] present as non-SOCS1 specific responses, even though both cytokines were released in greater quantities in the presence of *C. albicans*. We also find it intriguing that IL-10 production maintains similar levels under both *C. albicans* exposure and SOCS1 inhibition circumstances.

Through the production of distinct sets of cytokines and interaction with DCs, T cells undergo differentiation and then act as immune effectors (Th1, Th17, and Th2) or regulator (T_reg_) to govern innate and adaptive immunities. For example, Th1 and Th17 CD4 T cell subsets promote fungal clearance and confer protective immunity against diverse fungal pathogens [40, 41]. By contrast, Th2 responses have been found to play an inhibitory role in the immune response to fungal infection. In mice, reduction in SOCS1 gene expression in DCs leads to Th1-type hyper-responses [25]. In conjunction with high expressions of IL-12 and IFN-γ and low levels of IL-4, we find that SOCS1-silenced DCs induce a mixed lymphocyte reaction, characterized by the induction of Th1 cell proliferation (through IL-12 and IFN-γ) and the reduction in Th2 cell proliferation (driven by IL-4) when co-cultured with *C. albicans.* This result was validated in a parallel experiment using anti-IL-12 and anti-IFN-γ mAbs to suppress both cytokines on Th1 cells activation. Although Th17 cells are also important for activation of inflammation and recruitment of neutrophils in candidiasis, we did not observe the changes in Th17 population and IL-6 cytokines that is the traditional hallmark of Th17 proliferation.

We assume that the roles of SOCS1 are not limited to Th1-type responses during exposure to *C. albicans*. For example, the absence of total reversal with anti-IL-12 and anti-IFN-γ mAbs in this study suggests that something other than these two cytokines also contributes to Th1 differentiation. Interleukin 4 plays an important role in the differentiation of Th2 types, but its negative regulation for CD4^+^ Th1 cell differentiation has been well documented in the infectious process. We explain this excess Th1 response as a possible consequence of an insufficient amount of IL-4 in SOCS1 silencing DCs. Inhibition of Th1 differentiation by IL-6 via SOCS1 has also been reported, but non- specific IL-6 response in SOSC1 silencing DCs after exposure to *C. albicans* was seen in this study [42]. These results indicate that SOCS1 silencing mediates the effective immunity to *C. albicans* infection that favors Th1 cell-mediated response.

The control of DC migration is crucial for the initiation and maintenance of T cell-mediated immune responses. The expression of CCR7 on mature DCs has emerged as the master mediator of this highly complex migratory process [43]. When activated with inflammatory stimuli, the chemokine receptor CCR7 is up-regulated on DCs. Our findings show that *C. albicans* stimulates the expression of CCR7, which may be associated with the maturation of DCs. Since CCR7 expression levels were not affected by SOCS1 silenced DCs, this suggests that SOCS1 silencing enhances the maturation of DCs, but has no effect on the migration of DCs.

The current study investigates the effects of SOCS1 silencing on the maturation of bone marrow-derived DCs and the proliferation of T cells in response to *C. albicans*. DCs can also interact with B cells and NK cells, thus bridging the gap between innate and adaptive immunity. Although the question of how SOCS1-silenced DCs regulate other types of immune cells needs to be explored further, we speculate that SOCS1-silenced DCs favor the clearance of *C. albicans* and provide protective immunity against fungal infections. Obviously, these findings in vitro with BMDC need to be confirmed in vivo for antifungal immune response, and it is our intention to carry out this research in the near future.

In summary, this study shows that SOCS1 siRNA can be used to silence immunosuppressive molecules in DCs, promote the maturation of DCs in response to *C. albicans* in vitro, and induce competent Th1 cell responses to *C. albicans*, which accelerate the clearance of *C. albicans* and provide a protective immune response against *C. albicans*.

## Electronic supplementary material

Below is the link to the electronic supplementary material.

**Supplemental Fig.** **1.** SOCS1 siRNA interference did not affect the DC viability. DCs were cultured for 8 days and collected for the experiments. DCs were treated with SOCS1 siRNA for 2 h and co-cultured with or without *C. albicans* for 24 h. DCs were exposed to medium alone as control. The cells were stained with FITC-caspase-3 mAb and analyzed for caspase-3 activity by flow cytometry. (a, b and c) Data from one representative experiment of seven experiments are shown. (d) Cell viability was assessed by trypan blue exclusion assay. Data was expressed as mean ± SD of seven independent experiments. (TIFF 386 kb)

**Supplemental Fig.** **2.** SOCS1 siRNA infection reduced the SOCS1 protein and mRNA expression in DCs by western blot and qRT-PCR. Actin served as a loading control. (a,b) Western blotting was performed to determine the interference efficiency. Treatment with SOCS1-siRNA #2 caused an approximately 75 % decrease in SOCS1 expression quantified by densitometry. Levels of these mRNA in DCs were quantified by qRT-PCR, and normalized to β-actin levels as control. mRNA levels in uninfected control cells is 1. Data were mean from seven independent experiments. The bars represent SD. **p < 0.01. (TIFF 539 kb)
