# Supplementary figures and images for: Silenced suppressor of cytokine signaling 1 (SOCS1) enhances the maturation and antifungal immunity of dendritic cells in response to Candida albicans in vitro
Source: Immunol Res. 2014 Nov 9;61(3):206–18. doi: 10.1007/s12026-014-8562-8 (PMC4336647; doi:10.1007/s12026-014-8562-8)

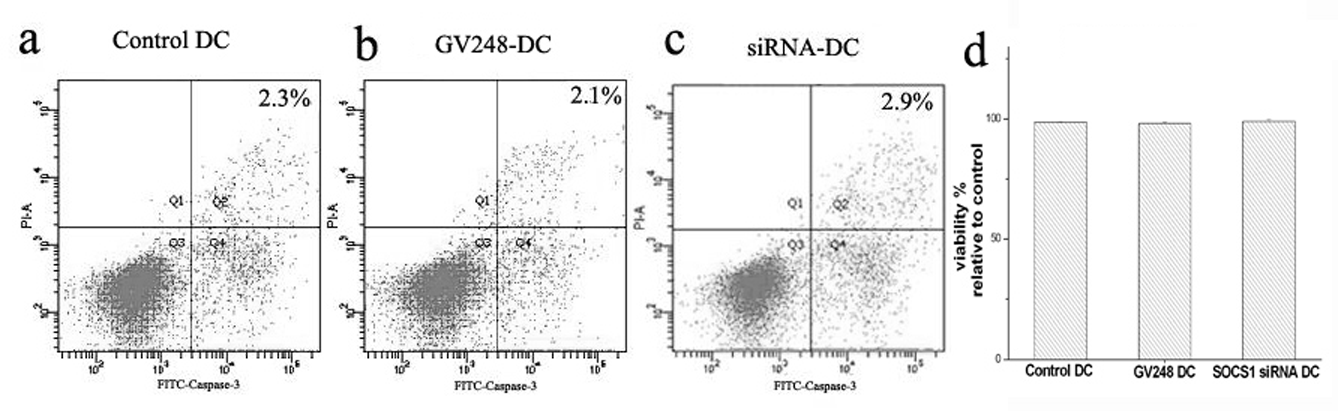

Supplement: Supplementary file 1 — Supplemental Fig. 1. SOCS1 siRNA interference did not affect the DC viability. DCs were cultured for 8 days and collected for the experiments. DCs were treated with SOCS1 siRNA for 2 h and co-cultured with or without C. albicans for 24 h. DCs were exposed to medium alone as control. The cells were stained with FITC-caspase-3 mAb and analyzed for caspase-3 activity by flow cytometry. (a, b and c) Data from one representative experiment of seven experiments are shown. (d) Cell viability was assessed by trypan blue exclusion assay. Data was expressed as mean ± SD of seven independent experiments. (TIFF 386 kb) [file 12026_2014_8562_MOESM1_ESM.tif]

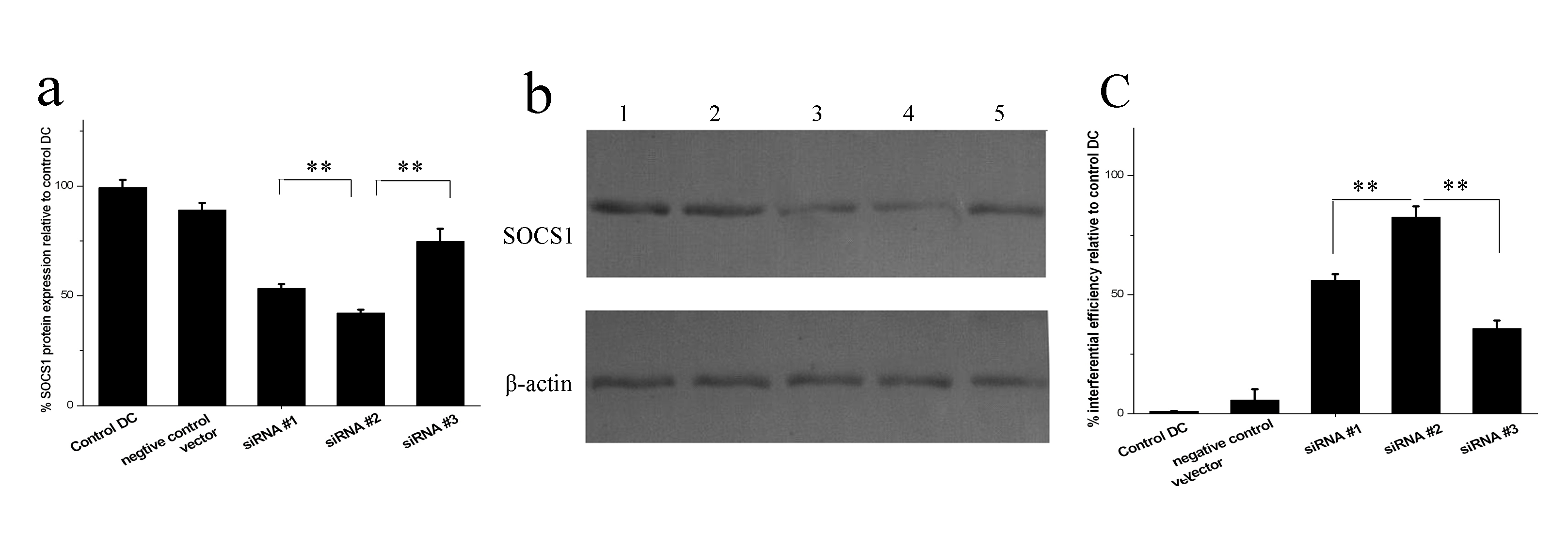

Supplement: Supplementary file 2 — Supplemental Fig. 2. SOCS1 siRNA infection reduced the SOCS1 protein and mRNA expression in DCs by western blot and qRT-PCR. Actin served as a loading control. (a,b) Western blotting was performed to determine the interference efficiency. Treatment with SOCS1-siRNA #2 caused an approximately 75 % decrease in SOCS1 expression quantified by densitometry. Levels of these mRNA in DCs were quantified by qRT-PCR, and normalized to β-actin levels as control. mRNA levels in uninfected control cells is 1. Data were mean from seven independent experiments. The bars represent SD. **p < 0.01. (TIFF 539 kb) [file 12026_2014_8562_MOESM2_ESM.tif]
